# Determinants and materno-fetal outcomes related to cesarean section delivery in private and public hospitals in low- and middle-income countries: a systematic review and meta-analysis protocol

**DOI:** 10.1186/s13643-016-0402-6

**Published:** 2017-01-14

**Authors:** Idrissa Beogo, Bomar Mendez Rojas, Marie-Pierre Gagnon

**Affiliations:** 1 École Nationale de Santé Publique, Ouagadougou, Burkina Faso; 2 Université Laval, 2325 rue de l’Université , Québec (Québec), G1V 0A6 Canada; 3 International Health Program, National Yang Ming University, 155 Sec 2, Linong St. 112, Taipei, Taiwan; 4 Faculty of Nursing, Université Laval, Ferdinand-Vandry Building, 1050 Avenue de la Médecine, Québec City (Québec), G1V 0A6 Canada

**Keywords:** Cesarean section, Materno-fetal outcome, Low- and middle-income countries, Systematic review

## Abstract

**Background:**

Despite the well-established morbidity, mortality, long-term effects, and unnecessary extra-cost burden associated with cesarean section delivery (CSD) worldwide, its rate has grown exponentially. This has become a great topical challenge for the international healthcare community and individual countries. Estimated at three times the acceptable rate as defined by the World Health Organization in 1985, the continued upward trend has been fuelled by higher income countries. Some low- and middle-income countries (LMICs) have now taken the lead, and the factors contributing to this situation are poorly understood. The expansion of the private healthcare sector may be playing a significant role. Distinguishing between the public and private hospitals’ role is critical in this investigation as it has not yet been approached. This review aims to systematically synthesize knowledge on the determinants of the CSD rate rise in private and public hospitals in LMICs and to investigate materno-fetal and materno-infant outcomes of CSD in perinatal period, between private and public hospitals.

**Methods/design:**

We will include studies published in English, French, Spanish, and Portuguese since 2000, using any experimental design, including randomized controlled trials (RCTs), non-RCTs, quasi-experimental, before and after studies, and interrupted time series. Outcomes of interest are the determinants of CSD and materno-fetal and materno-infant outcomes. We will only include studies carried out in private and public hospitals in LMICs. The literature searches will be conducted in the following databases: MEDLINE, Embase, CINAHL, Cochrane database, LILACS, and HINARI. We will also include unpublished studies in the gray literature (theses and technical reports). Using the two-person approach, two independent review authors will screen eligible articles, extract data, and assess risk of bias. Disagreements will be resolved through discussion with a third author. Results will be presented as structured summaries of the included studies. If possible, a meta-analysis will be conducted and, subsequently, an analysis for heterogeneity will be implemented.

**Discussion:**

The proposed systematic review of the CSD rate rise will provide up-to-date evidence in regard to differences in proportions, determinants, and materno-fetal and materno-infant outcomes in perinatal period, between private and public hospitals in LMICs. We believe that this knowledge synthesis will help to shed light on the evidence and support evidence-informed decision-making with a view to addressing the issue in LMICs.

**Systematic review registration:**

PROSPERO CRD42016036871

**Electronic supplementary material:**

The online version of this article (doi:10.1186/s13643-016-0402-6) contains supplementary material, which is available to authorized users.

## Background

Since 1985, the World Health Organization (WHO) has set the “ideal” rate of cesarean section delivery (CSD) at between 10 and 15% [1]. However, this has sounded like a liberalization mantra, prompting heated risk/benefit debates among scholars. There is a steep upward trend, and half of the world’s countries exceed the 15% level [2], with some reaching historically high rates. Once a concern of high-income countries [3, 4], the lead is now held by some low- and middle-income countries (LMICs), regardless of the continent [5, 6]: urban China (64.1%) [7], Colombia (43.4%), Dominican Republic (56.4%), Egypt (51.8%), Iran (47.9%), and Brazil (55.6%; 80% for second deliveries―when the first was by cesarean―and over 99% for third births―when the first two were by cesarean) [6, 8] to cite a few.

CSD is a major surgical procedure and far from being a harmless practice. It heightens the risk of major short- and long-term maternal morbidity and mortality [9–13], even in the case of elective CSD [14, 15]. A number of studies, including randomized control trials (RCTs) and systematic reviews, have underscored an increased likelihood of many short- and long-term adverse effects in mothers and babies [16, 17]. CSD upward growth has turned it into a great topical challenge for the international healthcare community and individual countries’ healthcare systems [18], raising conflicting policy-making priorities for governments. Nevertheless, higher CSD rate is not correlated to more favorable materno-fetal outcomes. On the contrary, a number of systematic reviews [19, 20] have shown that, apart from emergencies and some truly life-saving preventive circumstances with a sound cost-benefit ratio, CSD is clearly fraught with anomalous short-term immune responses (i.e., reduction of inflammatory marker expression in the newborn infant), a greater risk of developing immune diseases (such as asthma, allergies, DM type 1, celiac disease), a higher risk of cancer, and hematopoietic development problems. From an economic perspective, the cost of “unnecessary” CSDs performed worldwide is estimated at $2.32 billion, while the cost of “excess” ones amounts to approximately $432 million [2].

Within individual countries, variations in CSD rates and/or neonatal outcomes were found between different types of hospitals [21], in particular private versus public hospitals [22–24]. Some have hypothesized that this difference is associated with the hospital culture [25] or the payment scheme [26]. Other sources point to the role of supplier-side demand induction behavior [27, 28]. From a consumer perspective, some authors have suggested that women request to have an elective CSD in the absence of clinical indications [29–32]. However, McCourt et al.’s critical review [33] found that women’s preference for CSD varied only from 0.3 to 14%. This leaves room for other contributive factors, namely supply-side intervention.

Current practice of CSD is largely based on surgeon and institutional preference. One recent ecological study conducted in southern Asia and sub-Saharan Africa associated low CSD rate with poor access to the healthcare system, especially surgery; the wealthiest have higher CSD rates in resource-constrained settings [34]. Supply-side demand induction is often cited in the literature, and increased CSD rate seems to be related to professional convenience [35]. Hospital ownership matters and a meaningful difference are noted in CSD behavior between private and public hospitals [6]. Using a repeated measurement design, a significant difference in CSD decision was observed between private and public hospitals in Brazil [23]. Similarly, in Brazil, one of the two most significant contributing factors for CSD was giving birth under the private health system (OR = 4.3; 95%CI 2.3–9.0) [36]. Figures are even more striking in some other studies. In Peru, the CSD rate has climbed to 52.9% in private hospitals with a growth of 86.3% over a decade following the health reform [37]. In Brazil, the CSD rate among women attending private hospitals is significantly higher than the average, culminating up to 86.2%, with high toll paid by primiparous [38, 39].

## Rationale

Numerous systematic reviews have been conducted on CSD in LMICs, although to the best of our knowledge, none has approached the difference in CSD determinants and outcomes derived from public and private hospitals. The issue of CSDs is currently a global health concern. Its development in nascent private healthcare systems of LMICs constitutes an additional challenge. Therefore, this review will reveal what leads people to undergo a CSD in private or public health system, including medical and socioeconomic and demographic motives, whether the decision is shared or induced. It will further portray the medical outcomes derived for the dyad infant-mother perspective. Finally, systematically synthesizing evidence on the determinants and materno-fetal and materno-infant outcomes related to CSD is due, in order to up-to-date knowledge and support evidence-informed decision for making on appropriate policy options. Furthermore, the study focuses on LMICs.

## Research aims

This systematic review intends to investigate the determinants and the outcomes of CSD for the dyad materno-fetal and materno-infant in perinatal period. The research specific aims to be addressed are (1) to investigate the determinants of choosing a CSD between private and public hospitals in LMICs and (2) to investigate differences in materno-fetal and materno-infant outcomes deriving from CSD in perinatal period between private and public hospitals in LMICs.

## Methods

This review was registered in the International Prospective Register of Systematic Reviews (PROSPERO), registration number CRD42013004319, and follows the Cochrane Collaboration methods [40]. In this study, private providers are defined as “all organizations and individuals working outside the direct control of the state” [41]. They consist of for-profit (FP) and non-for-profit providers (NFP); the former are defined as benefit-focused and the latter include philanthropic medical institutions. Institutional stewardship is further considered to complete the definition of the private healthcare sector, because in LMICs, services delivered in the private sector may be publicly financed—commonly in NFP [42].

## Search strategy

Both published and unpublished studies will be examined. We will follow the Preferred Reporting Items for Systematic Reviews and Meta-Analyses (PRISMA) guide [43] for this review. The checklist of the PRISMA recommendations is included as an additional file (see Additional file 1). The search will be performed in English. An initial exploratory search through MEDLINE will be undertaken followed by an analysis of words taken from the titles, abstracts, keywords, and common descriptors. A concept plan will then be built with the identified keywords and descriptors to run the search in the following four databases: Ovid-Medline®, CINHAL, Cochrane database, and Embase. Two other freely available databases, LILACS and HINARI, which cover more LMIC research, will be screened. Unpublished studies will be sought from clinical trial registers, Google and Google Scholar, and conference proceedings. Finally, we will look through additional references from pertinent studies. Regardless of the setting, studies published in English, French, Spanish, and Portuguese will be included. Because of the vacuum in the literature on this topic for LMICs, the study will cover the period from January 1, 1990, to March 2016. The starting reference date (1990) was chosen to coincide with the adoption of the WHO’s 1985 recommendation setting the “ideal rate” for CSDs at 10 to 15%, assuming that a period of 5 years is a reasonable time lag for countries to implement this recommendation. The second reason was the expansion of improving CSD technology, which had started in the 1990s, in LMICs.

## Types of studies included

This review will include quantitative studies. The study design consists of any experimental study design, namely RCTs, non-RCTs, before and after quasi-experimental, and interrupted time series designs. Non-RCT studies will include case-control, cohort, and cross-sectional studies in which CSD is the primary outcome used.

## Inclusion/exclusion criteria

Countries are listed as LMICs based on the World Bank 2016 classification [44]. We will select studies that include medically prescribed planned and unplanned CSDs, done in accredited public or private healthcare settings, and elective CSDs. In addition, primiparous as well as multiparous, twin, and breech births will also be included. However, we will exclude (1) utilization of experimental CSDs, (2) stillbirth CSDs, and (3) outreach mass surgery (Table 1).

**Table 1:** MEDLINE concept plan, modified as needed for other databases

N0	Query
1	exp cesarean section /
2	(Ceasarean OR caesarian OR section OR cesarian OR “cesarean section”OR “caesarean section” OR CS OR C-S OR “C Section” OR C-Section OR “abdominal delivery” OR “abdominal deliveries” cesarean birth, cesarian birth, cesarean deliveries, cesarian deliveries, cesarean delivery, cesarian delivery)
3	1 OR 2
4	(LMIC OR LIC OR “low income” OR Africa OR Asia OR Latin OR “middle income” OR Afghanistan OR Gambia OR Niger OR Benin OR Guinea OR Rwanda OR “Burkina Faso” OR Burkina OR “Guinea Bisau” OR “Sierra Leone” OR Burundi OR Haiti OR Somalia OR Cambodia OR Korea OR Sudan OR “Central African Republic” OR centrafica OR Liberia OR Tanzania OR Chad OR Madagascar OR Togo OR Comoros OR Malawi OR Uganda OR Congo OR Mali OR Zimbabwe OR Eritrea OR Mozambique OR Ethiopia OR Nepal OR Armenia OR Indonesia OR Samoa OR Bangladesh OR Kenya OR “Sao Tomé and Principe” OR Bhutan OR Kiribati OR Senegal OR Bolivia OR Kosovo OR “Solomon Islands” OR Salomon OR “Cabo Verde” OR “Cape Verde” Kyrgyz* OR “Sri Lanka” OR Cameroon OR Lao OR Sudan OR Lesotho OR Swaziland OR “Cote Ivoire” OR “Ivory coast” OR Mauritania OR Syrian OR Djibouti OR Micronesia OR Tajikistan OR Egypt OR Moldova OR Timor OR “El Salvador” OR Salvador OR Morocco OR Ukraine OR Georgia OR Myanmar OR Uzbekistan OR Ghana OR Nicaragua OR Vanuatu OR Guatemala OR Nigeria OR Vietnam OR Guyana OR Pakistan OR Palestine OR Gaza OR Honduras OR “Papua New Guinea” OR Papouasie OR Papouasia OR Yemen OR India OR Philippines OR Zambia OR Albania OR Fiji OR Namibia OR Algeria OR Gabon OR Palau OR Samoa OR Grenada OR Panama OR Angola OR Iran OR Paraguay OR Azerbaijan OR Iraq OR Peru OR Belarus OR Jamaica OR Romania OR Belize OR Jordan OR Serbia OR Bosnia OR Herzegovina OR Kazakhstan OR “South Africa” OR Botswana OR Lebanon OR Lucia OR Brazil OR Libya OR Saint Vincent and the Grenadines OR Bulgaria OR Macedonia OR Suriname OR China OR Chinese OR Malaysia OR Thailand OR Colombia OR Maldives OR Tonga OR “Costa Rica” OR Marshall OR Tunisia OR Cuba OR Mauritius OR Turkey OR Dominica OR Mexico OR Turkmenistan OR Dominican OR Mongolia OR Tuvalu OR Ecuador OR Montenegro)
5	exp hospital /
6	hospital OR hospitals OR public OR private OR “health center” OR “health centers” OR “medical college” OR “medical colleges” OR “medical university” OR “medical universities”
7	5 OR 6
8	3 AND 4 AND 7

## Types of comparisons

This review will compare the CSDs performed in private versus public settings in LMICs. The study will extract data concerning the outcome (morbidities, mortalities, and medical complications) of CSDs as the primary outcome, as well as the motivators conducive to undergoing those CSD.

## Determinant and outcome measurement

This review will examine the key characteristics of studies that may determine the high CSD rate. It includes (1) the where (i.e., urban versus rural, as well as racial/cultural/ethnic background); (2) the who (i.e., practitioner characteristics, decision-making process); (3) the which (i.e., ownership, financing system, payment scheme, management system, and underlying philosophy); (4) consumer characteristics (fertile age women, sociodemographic, economic status); and (5) obstetrical patterns (i.e., parity, type, and quality of the pregnancy).

We will consider outcome measures as defined by the “Outcomes of interest to the Cochrane consumers & communication review group” [45]. Initially, primary outcomes consist of materno-fetal and materno-infant medical outcomes: (1) mortality, (2) morbidity, and (3) observed complications. The secondary outcomes include (1) participants’ knowledge/understanding of the risks of CSD procedures; (2) participants’ involvement in decision-making and their satisfaction with the decision taken; and (3) other outcomes: birth goal attainment, quality of life, self-efficacy, and social functioning.

## Data extraction and synthesis

We plan to use the *Rayyan systematic reviews web application* to explore and filter searches for eligible studies. Using the two-person approach, the first two authors, IB and BRM, will independently conduct the initial screening of all titles and abstracts captured in the databases to determine eligibility. Next, both results will be compared in order to identify any discrepancies. In this scenario, MPG, who is an experienced systematic review author and Cochrane group trainer, will intervene through a discussion to conclude this selection phase. Secondly, all pertinent references will be retained for a second round of assessment of the full texts, and disagreement will be resolved in the same fashion. If needed, we will contact authors of primary studies, either to request missing information or to clarify unclear data. Finally, the PRISMA flow diagram will report quantitative and qualitative information on the selection process.

## Assessment of methodological quality

Given the importance of quality assessment on the results of systematic reviews, the underlying methodological quality of studies, and the transferability of the results, we will assess each type of design separately. The Cochrane Risk of Bias tool [46] will be used to guide the assessment of RCT studies. Non-RCTS deemed eligible for inclusion will be appraised using the Joana Briggs Institute’s Qualitative Assessment and Review Instrument (JBI-QARI) [47], and Mixed Studies Review (MSR) [48] will be employed for mixed methods and quantitative observational designs.

Thus, all the authors will independently assess the final set of studies included according to a predefined risk of bias scoring key [46]:

- Selection bias: description of random components in the sequence generation
- Allocation concealment: how foreseeable the allocation of participants has proven to be
- Performance bias: assessment of measures blinding study participants and personnel from knowledge of the intervention a participant would receive
- Attrition bias: assessment of study participants’ withdrawal from the analysis
- Reporting bias: assessment of possible selection in expected or pre-specified outcomes, deriving from a systematic difference between reported and non-reported findings. This will be based on the existence of a trial protocol and whether the expected outcomes have been reported in a pre-specified way.
- Other sources of bias: sample size and power calculations of the trial are based on the reported outcome or confounding.

Finally, literature will be updated before the publication draft is issued. Any new, relevant studies will be added to the review results.

### Data analysis

Data from included studies will be extracted and stored in a customized Excel® spreadsheet. They will be structured according to the following items: the study setting, populations, study methods, information on the risk of bias assessed, and type of outcomes of significance in relation to the review question and specific objectives.

We anticipate that the number of studies will be sufficient to allow us to perform a meta-analysis. Therefore, data will be pooled to this end. Effect size will be presented as risk ratios (for categorical outcome) or weighted mean differences (for continuous data), all based on 95% confidence intervals and two-sided *p* values for each outcome. When the effects of clustering are not taken into account, we will adjust the standard deviations for the design effect. Heterogeneity will be assessed statistically using the standard Chi-square, I-squared statistic using subgroup analyses. The a priori planned subgroup analyses will consist of (1) ownership (FP vs. NFP vs. public providers); (2) population groups (i.e., socio-demographic factors, parity); and eventually (3) the type of hospital (i.e., district hospital, tertiary level private hospital). Private wards in publicly owned hospitals will be kept in the public provider category except if, apart from the amenities that distinguish them, they fall into a different clinical scheme applicable to their patients. I-squared value greater than 50% will be considered as indicative of substantial heterogeneity [40]. Findings from non-RCT studies will be presented in a narrative form.

Statistical analyses will be based on intention-to-treat and calculated using the Cochrane statistical package Review Manager.

## Discussion and conclusion

This systematic review aims to synthesize knowledge on CSD with respect to its epidemiology and materno-fetal and materno-infant outcome in function of hospital ownership in LMICs. As there is a wide array of motivations behind CSD practice, including medical and non-medical, personal, or system-driven culture, the present systematic review will assess the effect on a broad range of outcomes reported so far. An important body of literature does exist on CSD, including systematic reviews on more specific themes, but the “global epidemic” of CSDs, regardless of reported harms, is raising critical questions internationally. As the development of private hospitals is found to be among the drivers, this review will gather updated information from existing literature. Because this review aims to guide policy-making in LMICs, we will restrict its scope to evidence concerning LMICs, where the CSD rate is growing rapidly despite their fragile healthcare systems and economies.

## Additional file

Additional file 1: Preferred Reporting Items for Systematic review and Meta-Analysis Protocols (PRISMA-P) 2015 checklist: recommended items to address in a systematic review protocol.

## Abbreviations

CSD: Cesarean section delivery
FP: For-profit
LMIC: Low- and middle-income countries
NFP: Non-for-profit
PRISMA: Preferred Reporting Items for Systematic Reviews and Meta-Analyses
PROSPERO: International Prospective Register of Systematic Reviews
RCT: Randomized control trial
WHO: World Health Organization
